# MicroRNA Profiling of Exosomes Derived from Red Blood Cell Units: Implications in Transfusion-Related Immunomodulation

**DOI:** 10.1155/2019/2045915

**Published:** 2019-06-13

**Authors:** Haobo Huang, Jinfeng Zhu, Liping Fan, Qiuyan Lin, Danhui Fu, Biyu Wei, Shijin Wei

**Affiliations:** ^1^Department of Blood Transfusion, Fujian Medical University Union Hospital, Gulou District, Fuzhou City, Fujian Province 350001, China; ^2^Department of Oncology, Quanzhou First Hospital Affiliated to Fujian Medical University, Quanzhou City, Fujian Province 362000, China; ^3^Department of Hematology, Fujian Medical University Union Hospital and Fujian Institute of Hematology, Gulou District, Fuzhou City, Fujian Province 350001, China

## Abstract

**Purpose:**

To elucidate the microRNAs existent in exosomes derived from stored red blood cell (RBC) unit and their potential function.

**Materials and Methods:**

Exosomes were isolated from the supernatant derived from stored RBC units by sequential centrifugation. Isolated exosomes were characterized by TEM (transmission electron microscopy), western blotting, and DLS (dynamic light scattering). MicroRNA (miRNA) microarray was performed to detect the expression of miRNAs in 3 exosome samples. Results revealed miRNAs that were simultaneously expressed in the 3 exosome samples and were previously reported to exist in mature RBCs. Functions and potential pathways of some detected miRNAs were illustrated by bioinformatic analysis. Validation of the top 3 abundant miRNAs was carried out by qRT-PCR (quantitative reverse transcription‐polymerase chain reaction).

**Results:**

TEM and DLS revealed the mean size of the exosomes (RBC-derived) as 64.08 nm. These exosomes exhibited higher abundance of short RNA than the long RNA. 78 miRNAs were simultaneously detected in 3 exosome samples and mature RBCs. Several biological processes might be impacted by these miRNAs, through their target gene(s) enriched in a particular signalling pathway. The top 3 (abundant) miRNAs detected were as follows: miR-125b-5p, miR-4454, and miR-451a. qRT-PCR revealed higher abundance of miR-451a than others. Only miR-4454 and miR-451a abundance tended to increase with increasing storage time.

**Conclusion:**

Exosomes derived from stored RBC units possessed multiple miRNAs and, hence, could serve various functions. The function of exosomes (RBC-derived) might be implemented partly by the predominantly enriched miR-451a.

## 1. Introduction

Clinically, allogeneic red blood cell (RBC) transfusion is an important therapeutic approach. However, several studies revealed that RBC transfusion was associated with poor prognosis in some cancer types and critically ill patients or that it affected the immune system of patients who needed chronic transfusions [1–6].

Transfusion experts suggested that transfusion-related immunomodulation (TRIM) of blood recipients might shed some light and help in understanding these phenomena [7, 8]. The presence of “something” has been reported in the blood suspension, during storage, which participated in abnormal functioning of immune cells such as T-cells and monocytes* in vitro* [7–11]. Samples drawn from the blood recipients exhibited significant abnormality in the quantity or function of cells of the immune system* in vivo* [3, 4, 6]. However, the mechanisms underlying these phenomena were not elucidated and understood clearly.

Exosomes are membrane-derived vesicles, with a size range of 20–200 nm. They are the products of exocytosis; they contain DNA, coding or noncoding RNAs (ncRNAs), and protein fragments that are secreted by their parental cell and can be taken into the recipient cells. Exosomes are reported to carry out several physiological and pathophysiological functions during carcinogenesis and immunomodulation in both* in vitro* and* in vivo* conditions [12–15]. ncRNAs of the exosomes have been proved to play an important role in regulation of these procedures [14, 15].

Danesh et al. in their study have revealed that exosomes derived from RBC units could potentiate T-cell survival and mitogen-induced proliferation through antigen presenting cells (APCs), eventually contributing to TRIM [9]. The “things” that exosomes possessed and transferred to the APCs still remain unidentified. Doss et al. demonstrated that mature erythrocytes possess a diverse repertoire of miRNAs which are relatively more abundant than the long RNAs [16]. Therefore, we performed a series of experiments* in vitro* to illustrate the miRNAs (that exosomes derived from the RBC units) and their implicated functions.

## 2. Materials and Methods

### 2.1. Study Samples

Ten bags of prestorage leukoreduced RBC units with anticoagulant (ACD-A) and stabilizing mixture (8.0 g/L citric acid monohydrate, 22.0 g/L sodium citrate monohydrate, and 24.5 g/L glucose monohydrate) were supplied by the Blood Center of Fujian Province, China. A written consent was also obtained from the Fujian Provincial Health Commission and the study protocols were approved by the ethics committee of the Fujian Medical University Union Hospital, China.

### 2.2. Isolation and Purification of Exosome

We extracted 15 mL of leukoreduced RBC suspension from each bag after 7, 14, 21, 28, and 35 days of storage. This accounted for a total of 50 suspension samples. Supernatants were obtained by an initial centrifugation at 3,000* g *for 10 min, followed by a brief centrifugation, performed with 0.22 *μ*m filters at 750* g *for 2 min. Supernatants were stored at -80°C. To extract the exosomes, supernatants were first thawed and then were subjected to sequential centrifugation at 13,000* g* for 30 min (at 4°C), followed by centrifugation at 100,000* g* for 60 min (at 4°C) (Himac CS150GXII, HITACHI, Japan). Exosome pellets obtained were resuspended in 0.1 mL of PBS (phosphate buffered saline) for TEM and particle size analysis, in 0.1 mL RIPA buffer for protein quantification and western blotting, and in 1 mL of TRIzol reagent (Thermo Fischer Scientific, USA) for RNA quantification, microarray, and qRT-PCR.

### 2.3. Detection of Exosomes Derived from RBC Units

Exosomes were placed as a 20 *μ*L drop on a 2 *μ*m copper grid. The drop was dried for 5 min at room temperature and the excess liquid from the grid edge was drained with the help of a filter paper. The grid was subsequently placed onto a drop of 2% phosphotungstic acid (pH 7.0) for 30 sec, and the excess liquid was drained off as above. The grid, after drying for 5 min, was analysed using a TEM (Model H-7650, Hitachi, Japan).

Particle size distribution was analysed by Zetasizer Nano ZS90 (Malvern Panalytical, UK). Samples were diluted in PBS in the ratio 1:20, before loading them manually into the sample chamber. Three videos of 60 sec each were recorded of each sample. Data was analysed by using DTS v5.10 software (Malvern Panalytical, UK). Results were displayed as particle size distribution.

Purified exosomes and RBCs were treated with RIPA buffer. Protein concentration was estimated by Nanodrop® ND-1000 (Thermo Fischer Scientific, USA). Total protein was separated on a 7.5–12% sodium dodecylsulphate polyacrylamide gel electrophoresis (SDS-PAGE) gel which was later transferred onto PVDF membranes (Millipore, USA). Membranes were blocked for 2 hr with 5% fat-free milk dissolved in tris-buffered saline containing 0.05% Tween-20 (TBST). This was followed by an overnight incubation at 4°C with primary antibodies against TSG101 (Abcam, USA, 1:1000), CD63 (Abcam, 1:1000), and Calnexin (Abcam, 1:2000). Membranes were washed thrice with TBST, followed by an incubation at room temperature for 1 hr with the corresponding anti-mouse or anti-rabbit HRP- (horseradish peroxidase-) conjugated secondary antibodies. Subsequently, the membranes were washed and the signals were visualized and captured with SuperSignal West Dura Substrate (Pierce, USA) and ChemiDoc™ XRS+ system (Bio-Rad, USA), respectively.

### 2.4. RNA Isolation and miRNA Expression Profiling

Total RNA was extracted using TRIzol reagent and purified with RNeasy minikit (QIAGEN, German) according to the manufacturer's instructions. The quantification and quality estimation of the total extracted RNA were carried out by Nanodrop® ND-1000. Integrity of the total RNA was analysed by denaturing agarose gel electrophoresis.

miRNA profiling of 3 exosome samples (RBC-derived, 14 day storage) was performed by miRCURY LNATM microRNA array kit (Exiqon, Denmark) following the standard protocols at the Aksomics biotech Co., Ltd. (Shanghai, China). After the quality control, miRCURY™ LNATM microRNA Array Hy3™/Hy5™ Power labelling kit (Exiqon; Vedbaek, Denmark) was used for miRNA labelling according to the manufacturer's guideline. The Hy3™-labelled samples were hybridized on the miRCURYTM LNA miRNA Array (v.19.0) (Exiqon) after stopping the labelling process, by following the manual. Finally, the slides were scanned using a GenePix® 4000B microarray scanner (Axon Instruments, Foster City, CA) and the scanned images were imported into GenePix® Pro 6.0 software (Axon Company, Beijing, China) for grid alignment and data extraction.

### 2.5. Bioinformatic Analysis

Candidate miRNAs which (1) existed in all the three samples detected by miRNA microarray and (2) existed in RBC previously described [16] were selected. FunRich software (version 3, http://www.funrich.org) was used for enrichment analysis of the candidate miRNAs and the top 10 abundant miRNAs were selected for miRNA-mRNA interaction analysis. miRNA target prediction was performed by TargetScan 7.2 (http://www.targetscan.org/vert_72/). Predicted gene(s) with cumulative weighted “context++ score” >-0.5 was selected for miRNA-mRNA analysis. Cytoscape software (https://cytoscape.org/) was used to obtain a network of miRNAs and mRNAs which displayed the relationship between miRNAs and its targets.

### 2.6. Validation of Candidate miRNAs by qRT-PCR

The top 3 abundant miRNAs that fulfilled the above listed criteria were validated by qRT-PCR. Total RNA of 50 exosome samples was extracted (described above). The first-strand cDNA was synthesized by different reverse transcription primers using M-MLV reverse transcriptase (Epicentre, USA), which was then used for qRT-PCR analysis (Table 1). qRT-PCR was performed for each sample in triplicate in a QuantStudio 5 Real-time PCR System (Applied Biosystems, USA) by following the manufacturer's instructions and using different primers (Table 2). The primers were synthesized by BIOLIGO Biotech (Shanghai, China). Relative expression of the top 3 miRNAs was assessed the 2^−ΔCt^ method with U6 snRNA as a housekeeping control.

### 2.7. Statistical Analysis

The results were expressed as mean±sd (standard deviation), and the rest of the statistical data was analysed and visualized by Prism 6.0 (GraphPad Software, USA). The significance of RNA quantities and qRT-PCR validation of miRNAs among exosomes (derived from RBC units stored for different time periods) was evaluated with one-way ANOVA, followed by paired t-tests. p<0.05 was considered to be significant throughout.

## 3. Results

### 3.1. Characterization of Exosomes Derived from RBC Units

**Figure 1:**
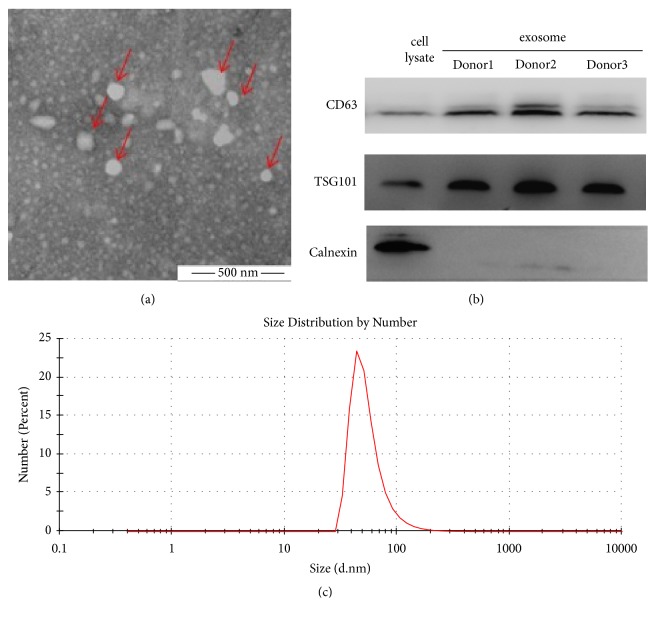
Identification of the exosomes derived from RBC units. (a) Morphology of exosomes, as revealed by TEM. (b) Exosome-specific markers (positive: CD63 and TSG101; negative: Calnexin) as detected by western blotting. (c) Particle size distribution by DLS.

Exosomes isolated from RBC units were analysed by TEM, DLS, and western blotting for morphology, size distribution, and specific immunological markers. TEM revealed the exosomes as cup-shaped morphologically (Figure 1(a)). Western blotting revealed CD63 and TSG101 to be present (positive) in exosomes and parental RBCs. However, Calnexin was negative in exosomes, but positive in parental RBCs (Figure 1(b)). Size of the exosomes as per DLS was 64.08±7.56 nm in diameter (Figure 1(c)).

### 3.2. RNA Content of Exosomes Derived from RBC Units

**Figure 2:**
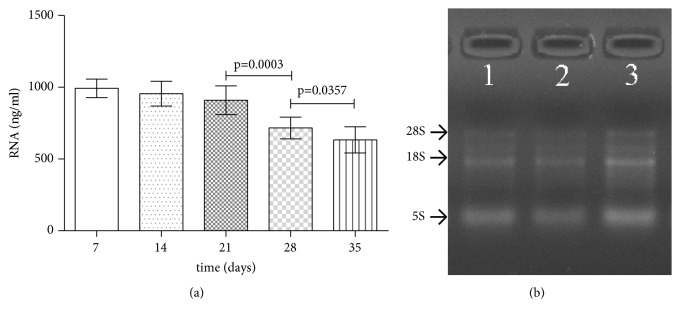
Quantification and integrity of RNA contents of exosomes derived from RBC units. (a) The quantification of the total RNA in 50 exosome samples (as detected by Nanodrop® ND-1000). (b) Estimation of total RNA integrity in 3 exosome samples (Nos. 47603, 49603, and 42420) by denaturing agarose gel electrophoresis.

No significant difference was observed in RNA quantities of exosomes obtained from RBC units stored for 7, 14, and 21 days (992.7±20.25 ng/mL, 956.0 ± 27.24 ng/mL, and 909.1±31.51 ng/mL, respectively, p=0.16). However, a reduction in RNA quantities of exosomes derived from RBC units stored for 28 and 35 days (716.4±24.04 ng/mL and 633.5±28.87 ng/mL, p<0.0001) was observed (Figure 2(a)). Denaturing agarose gel electrophoresis aided the visualization of all the bands of RNA, with thick bands corresponding to short RNA (Figure 2(b)).

### 3.3. miRNA Expression Profiling of the Exosomes Derived from RBC Units

**Figure 3:**
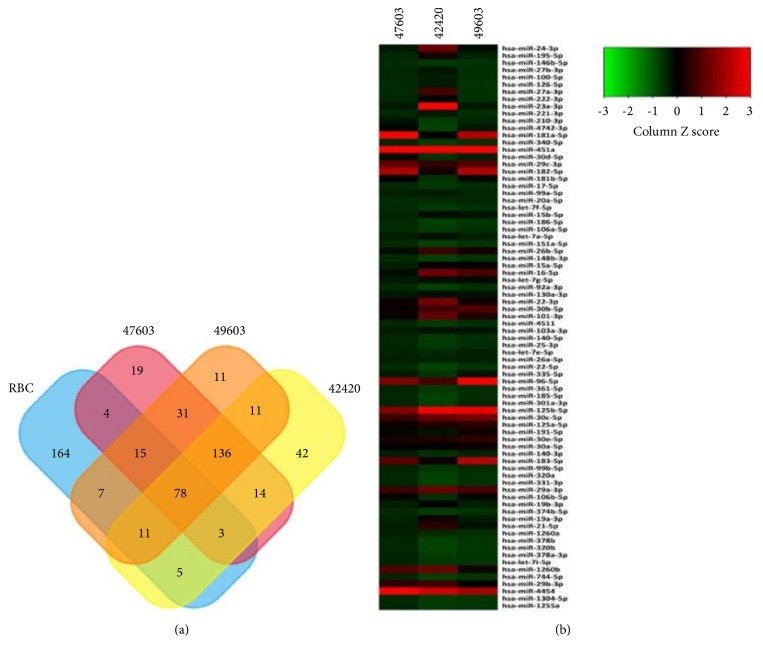
miRNA expression profiling of exosomes (RBC-derived). (a) Venn diagram exhibiting the miRNAs that are simultaneously expressed in all of 3 exosome samples and are also present in mature RBCs. (b) Heat map revealing the abundance of 78 miRNAs (simultaneously expressed in all 3 exosome samples) as screened by three microarray assays.

Microarray data of the 3 exosome samples (Nos. 47603, 49603, and 42420) derived from RBC units (stored for 14 days) were submitted and uploaded to GEO dataset (Series GSE95512). Previous studies have reported 287 miRNAs in mature RBCs [16]. However, our study revealed a total of 78 miRNAs in all 3 samples (Figure 3(a)), wherein some showed higher abundance than the others (Figure 3(b)).

### 3.4. Bioinformatic Analysis

**Figure 4:**
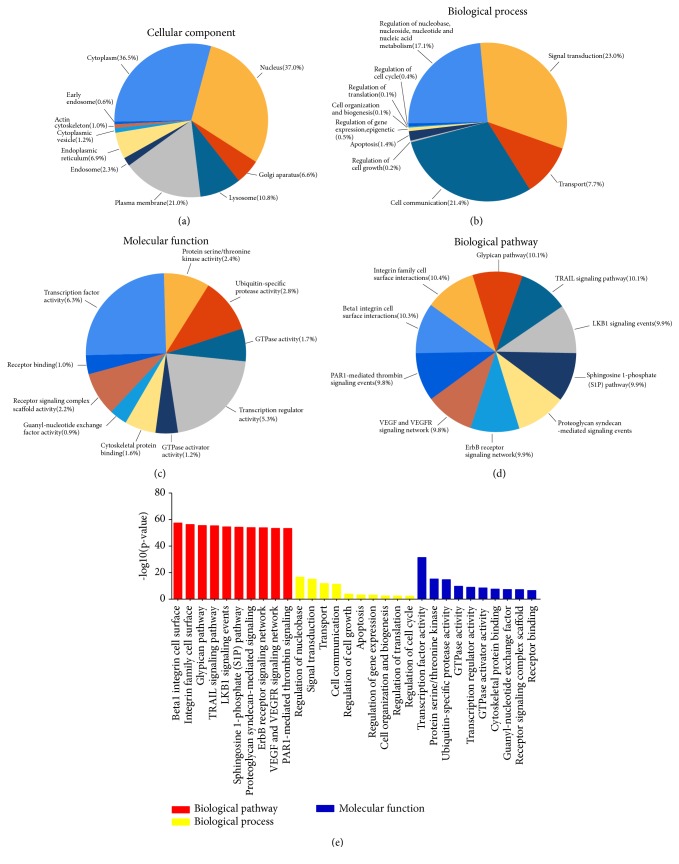
Enrichment analysis of the top 10 abundant miRNAs. (a–c, e) The 10 most enriched categories and the enrichment scores (-log 10(p value), p < 0.05) in Cellular Component, Biological Process, and Molecular Function were shown. (d) The top 10 important pathways.

**Figure 5:**
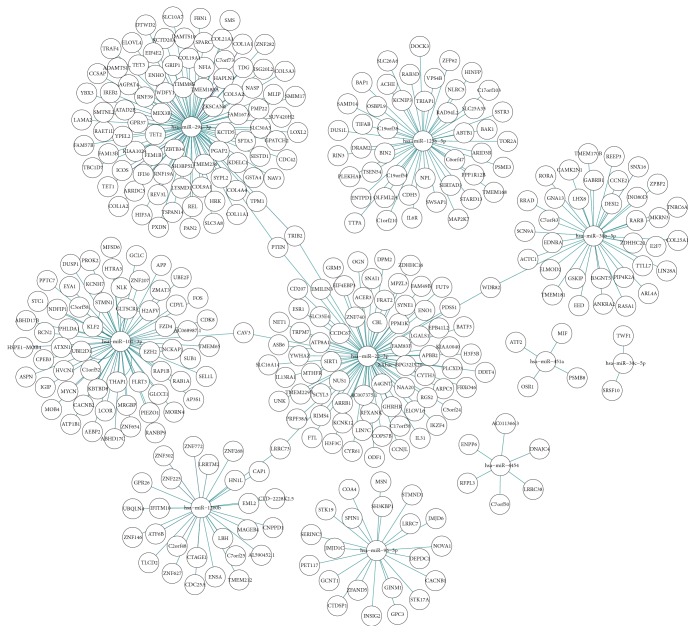
The top 10 abundant miRNAs, targeted genes, and networks.

For enrichment analysis, all exosomal miRNAs were selected. Ten most enriched categories in Cellular Component, Biological Process, and Molecular Function with the top 10 important pathways are shown in Figure 4. Target genes of the top 10 abundant miRNAs were predicted by the software TargetScan 7.2 (http://www.targetscan.org/vert_72/).  Figure 5 illustrates the top 10 abundant miRNAs, their target genes, and networks.

### 3.5. Validation of miRNAs by qRT-PCR

**Figure 6:**
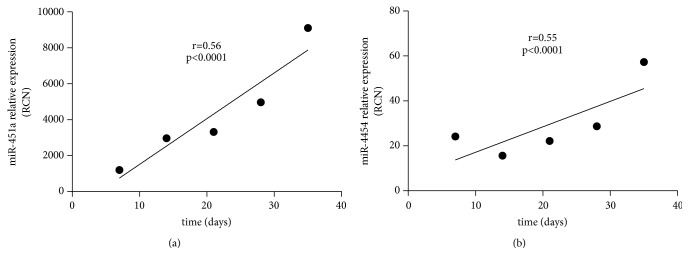
Analysis of correlation between expression of exosomal miR-451a (a), miR-4454 (b), and storage time.

Top 3 abundant miRNAs out of 50 exosome samples were selected for validation study using qRT-PCR (Table 3). The results reflected no significant difference in miR-125b-5p expression among RBC units with different storage time periods (p=0.14). However, a significant increase in the expression of miR-125b-5p was noted after 21 days of storage. After 35 days of storage, miR-125b-5p expression attained the peak level. Apart from this, a substantive difference was recorded in miR-4454 and miR-451a expression among the RBC units at different storage time (p=0.0004 and 0.0123, respectively). After 14 days of storage, miR-4454 expression increased with the extension in storage time (r=0.55, p<0.0001; Figure 6(a)). On the other hand, abundance of miR-451a did not positively correlate with the storage time (r=0.56, p<0.0001, Figure 6(b)). However, an increase in expression of miR-451a was noted with the extension of storage time. Expression levels of miR-4454 and miR-451a were observed to be highest in RBCs after 35 days of storage.

## 4. Discussion

With the development of new medical technology, like neoadjuvant chemoradiotherapy, the possibility of allogeneic RBC transfusion is increasing [17]. Allogeneic RBC transfusion can improve the outcome in recipients by establishing blood volume, improving blood perfusion, and changing the gut microbiome [2, 18–25]. However, allogeneic RBC transfusion can also expose recipients to a chance of immunosuppression or poor survival.

Experts attribute these phenomena to suppression of function of immune cells, including immune effector cells and helper cells [3, 4, 7–11]. Up to date, several researchers have reported that soluble biological mediators (cytokines, growth factors) and subcellular components (extracellular vesicles (EVs)) present in the supernatant of blood products can affect the biological behaviour of immune cells and tumour cells* in vitro*. This, in turn, leads to immunosuppression or poor survival of the recipient* in vivo *[3, 4, 7–11, 26].

Exosomes are microvesicles with a lipid-bilayer, which are secreted by almost all cell types and aid in mediating cell-to-cell communication. Recently, exosomes (secreted by diverse cell types) were reported to play various roles in physiological processes—such as cell development or differentiation—and pathophysiological processes, such as carcinogenesis, metastasis, drug resistance, and immunomodulation by different mechanisms [8, 12–15, 27, 28]. However, the role and mechanisms of exosomes (RBCs-derived) in transfusion-related immunomodulation await clear elucidation.

Secretion and contents of exosomes are regulated by different microenvironments [13, 27, 29, 30]. The contents of exosomes (RBC-derived), stored in ACD-A solution, are still unknown. We have, for the first time, reported the presence of exosomes in the supernatant of RBC units. In recent years, exosomal ncRNAs are documented as an important mediator in regulating intercellular communication. In the present study, we noted the RNA contents of exosomes and found them to decrease after 21 days of storage. Exosome degradation was proposed to contribute to this phenomenon. Also, the results of gel electrophoresis revealed the abundance of short RNA compared to the long RNA, which was similar to that of mature RBC as reported by Doss et al. [16]. Three exosome samples were selected to estimate the miRNA content by using miRNA microarray. Although previous studies have reported the presence of 287 miRNAs in mature RBCs, the present study revealed the presence of 78 miRNAs in the 3 exosome samples and that only some of them had relatively high abundance. It was proposed that selective enrichment of miRNAs might lead to higher abundance of some miRNA types in the exosome due to the presence of a special EXOmotif of miRNAs and miRNA sorting proteins such as hnRNPA2B1 in the parental RBCs [31].

To identify the potential function of the exosomal miRNAs, enrichment analysis of 78 miRNAs was performed. Various biological processes including signal transduction and nucleotide metabolism might be impacted by these miRNAs, through their target gene(s), most of which were enriched in several signalling pathways and were localized to the nucleus, cytoplasm, and plasma membrane.

Subsequently, we estimated the expression levels of top 3 abundant miRNAs: miR-125b-5p, miR-451a, and miR-4454, using a qRT-PCR. The results revealed the presence of these 3 miRNAs in all exosome samples (with different storage periods). During storage, exosomal miR-125b-5p was the least abundant among the top 3 miRNAs. Also, its expression level did not change with increasing storage time; however, it attained the peak level after storage of 35 days. A positive correlation could not be deduced between the abundance of exosomal miR-4454 and the storage time, although a tendency of increase in expression could be noted with the extending storage time. Similar results were obtained in case of exosomal miR-451a. In the present study, we noted that miR-451a was highly abundant among the top 3 miRNAs at each storage time. However, none of the 3 miRNAs could serve as a biomarker for predicting storage lesions and monitoring the quality of RBC units. Although mature RBCs can not generate new RNA molecules and exosomal RNA molecules are possibly degraded with the extending storage time, we can find increasing of these 3 miRNAs during storage and attribute this phenomenon to the changes in microenvironment of stored RBCs which were previously reported [32], leading to the changes in contents of exosomes [13, 27, 29, 30]. However, high abundance of exosomal miR-451a may mark it as an important regulatory miRNA in the recipients. Till date, several researches have demonstrated that miR-451a could inhibit the proliferation and differentiation of benign and malignant tumour cell and also affect the chemosensitivity of the tumour cells. Moreover, miR-451a in extracellular vesicles has been reported to influence the functions of immune cells, such as macrophages and dendritic cells [28, 33–36]. Hence, in the present study the predominantly enriched miR-451a in exosomes may be speculated to act as an important mediator in TRIM.

However, our study has some limitations. Since we validated only the top 3 miRNAs, several additional miRNAs still need to be validated. Moreover, the function elucidation of exosomal miR-451a needs to be carried out* in vitro* and* in vivo*.

## Figures and Tables

**Table 1:** List of reverse transcription primers used in first-strand cDNA synthesis.

Gene	RT primer
U6	5' CGCTTCACGAATTTGCGTGTCAT3'
miR-4454	5' GTCGTATCCAGTGCGTGTCGTGGAGTCGGCAATTGCACTGGATACGACTGGTGC3'
miR-451a	5' GTCGTATCCAGTGCGTGTCGTGGAGTCGGCAATTGCACTGGATACGACAACTCA3'
miR-125b-5p	5' GTCGTATCCAGTGCGTGTCGTGGAGTCGGCAATTGCACTGGATACGACTCACAA3'

**Table 2:** List of primers used in qRT-PCR.

Gene	Primers	Annealing temperature(°C)	Product length (bp)
U6	F:5'GCTTCGGCAGCACATATACTAAAAT3' R:5'CGCTTCACGAATTTGCGTGTCAT3'	60	89
miR-4454	GSP:5'GGCACGATCCGAGTCACG3' R:5'GTGCGTGTCGTGGAGTCG3'	60	62
miR-451a	GSP:5'GGGGGAAACCGTTACCATTAC3' R:5'GTGCGTGTCGTGGAGTCG3'	60	65
miR-125b-5p	GSP:5'GCTCCCTGAGACCCTAAC3' R:5'GTGCGTGTCGTGGAGTCG3'	60	62

**Table 3:** Expression data of the 3 validated miRNAs in exosomes (RBC-derived, stored for different time periods).

miRNA	Storage time (in days)				
	7	14	21	28	35
miR-125b-5p	0.22±0.06	0.26±0.04	0.18±0.03	0.32±0.07	0.39±0.06
miR-4454	24.14±5.43	15.63±1.62	22.19±2.19	28.70±2.54	57.27±8.66
miR-451a	1202±374.6	2970±480.5	3322±764.2	4968±478.4	9108±2437

miRNA expression is expressed as relative copy number (RCN) using the value 2^−∆Ct^.

## Data Availability

Some data used to support the findings of this study are available from GEO dataset (Series GSE95512). Other data used to support the findings of this study are included within the article. Data are available from the corresponding author (Liping Fan: fanliping1982@163.com; Qiuyan Lin: lqyjmz@163.com) for researchers who meet the criteria for access to confidential data.
